# Evaluation of nefrotoxicity by tacrolimus and micophenolate mofetil associated with kidney ischemia and reperfusion: experimental study in rats

**DOI:** 10.1590/0100-6991e-20223233-en

**Published:** 2022-08-01

**Authors:** ALEXANDRE CAVALHEIRO CAVALLI, LUIS FERNANDO MACENTE SALA, JULIO SLONGO, ROGERIO DE FRAGA, RENATO TAMBARA

**Affiliations:** 1 - Universidade Federal do Paraná, Urologia - Curitiba - PR - Brasil; 2 - Hospital Nossa Senhora das Graças, Urologia - Curitiba - PR - Brasil; 3 - Kadlec Clinic, Urology - Richland - Washington - Estados Unidos

**Keywords:** Tacrolimus, Oxidative Stress, Immunology, Reperfusion, Tacrolimo, Estresse Oxidativo, Imunologia, Reperfusão

## Abstract

**Objective:**

to evaluate the renal toxicity caused by tacrolimus and mycophenolate mofetil (MMF) in a single kidney ischemia and reperfusion model.

**Method:**

experimental study using Wistar rats, submitted to right nephrectomy and left renal ischemia for 20 minutes, separated into groups in the postoperative period (PO): 1) Control (nonoperated); 2) Sham (operated, without PO drug); 3) TAC0.1, TAC1 and TAC10, tacrolimus administered PO at doses of 0.1mg/kg, 1mg/kg and 10mg/kg via gavage, respectively; 4) MMF, administered mycophenolate mofetil 20mg/kg; 5) MMF/TAC1 and MMF/TAC0.5, with an association of mycophenolate mofetil 20mg/kg and tacrolimus 1mg/kg and 0.5mg/kg, respectively. They were killed on the 14^th^ PO and the kidney was removed for tissue oxidative stress analysis, by the dosage of reduced glutathione (GSH), lipoperoxidation (LPO) and protein carbonylation (PCO), and histological analysis by glomerular stereology (Glomerular volume density, Numerical density glomerular and mean glomerular volume). Renal function was evaluated by the measurement of serum creatinine and urea.

**Results:**

both drugs caused alterations in renal function, and the toxicity of tacrolimus was dose-dependent. Subacute toxicity did not show significant glomerular histological changes, and there was renal and compensatory glomerular hypertrophy in all groups except TAC10*.*

**Conclusion:**

Both drugs cause changes in renal function. Glomerular morphometry and stereology showed negative interference of immunosuppressants during compensatory glomerular hypertrophy.

## RESUMO

**Objetivo:**

avaliar a toxicidade renal causada pelo tacrolimus e micofenolato mofetil (MMF) em um modelo de isquemia e reperfusão de rim único.

**Método:**

estudo experimental utilizando ratos Wistar, submetidos á nefrectomia direita e isquemia renal esquerda por 20 minutos, separados em grupos no pós- operatório (PO): 1) Controle (não operados); 2) Sham (operados, sem droga PO); 3) TAC0.1, TAC1 e TAC10, administrado tacrolimus no PO nas doses 0,1mg/kg, 1mg/kg e 10mg/kg via gavagem, respectivamentae; 4) MMF, administrado micofenolato mofetil 20mg/kg; 5) MMF/TAC1 e MMF/TAC0.5, com associação de micofenolato mofetil 20mg/kg e tacrolimus 1mg/kg e 0,5mg/kg, respectivamente. Foram mortos no 14º PO e retirado rim para análise do estresse oxidativo tecidual, pela dosagem de glutationa reduzida (GSH), lipoperoxidação (LPO) e carbonilação de proteínas (PCO), e análise histológica por estereologia glomerular (Densidade de volume glomerular, Densidade numérica glomerular e Volume glomerular médio). Foi avaliada função renal pela dosagem de creatinina e uréia séricas.

**Resultados:**

ambas drogas provocaram alteração na função renal, sendo a toxicidade do tacrolimus dosedependente. A toxicidade subaguda não mostrou alterações histológicas glomerulares significativas, sendo que houve hipertrofia renal e glomerular compensatória em todos os grupos exceto em TAC10.

**Conclusão:**

Ambas drogas provocam alteração na função renal. A morfometria e a estereologia glomerular mostraram interferência negativa dos imunossupressores durante a hipertrofia glomerular compensatória..

## INTRODUCTION

Rejection in the acute phase is still the biggest cause of kidney graft loss^1^. In recent years, Tacrolimus and Mycophenolate Mofetil have played an important role in post-transplant therapy, being widely used as central agents in current immunosuppression regimens, despite being potentially harmful to kidney tissue^2^. The exact mechanism of renal cell injury is not known^3^. Therefore, there is a need to deepen the knowledge about the renal effects caused by immunosuppression with potentially nephrotoxic drugs, also considering factors related to the kidney transplant procedure and organ manipulation.

In this study, we evaluated the subacute nephrotoxicity of the immunosuppressive drugs Tacrolimus and Mycophenolate Mofetil in rats submitted to a process of renal ischemia and reperfusion, demonstrating negative interference of immunosuppressants during compensatory glomerular hypertrophy.

## METHODS

The experiment was evaluated and approved by the Ethics Committee for the Use of Animals at the 6^th^ Ordinary Meeting of the CEUA of 2009 (07/10/2009, certificate 386 b), with correction and new approval of the changes in the Ordinary Meeting 03/2011 (04/05/2011). We observed the ethical principles in animal experimentation recommended by the Brazilian College of Animal Experimentation (COBEA) and the requirements established in the Guide for the care and use of experimental animals^4^.

The experimental phase took place in different stages, with the surgical procedures and histological and stereological analyzes carried out in the Department of Anatomy, and the oxidative stress tests, in conjunction with the Department of Cell Biology, both from the Biological Sciences Sector of the Federal University from Paraná.

We used male Wistar rats (Rattus norgicus albinus, Rodentia, Mammalia), with a mean age of nine weeks, mean weight ranging between 221 and 254 grams, kept in cages with the same environmental conditions, light/dark cycle of 12 hours, receiving water and food ad libitum during the whole period of the experiment^5^.

We randomly divided the animals into eight groups. Except for the animals of the Control Group, all underwent median laparotomy following the aseptic technique, right nephrectomy, hot renal ischemia by clamping the left renal pedicle for 20 minutes, renal reperfusion after declamping the vascular pedicle, and cystotomy followed by cystorrhaphy. For the surgical procedure, the animals were submitted to an intramuscular injection of diazepam (5mg/kg) and atropine (0.5mg/kg) and anesthetized with an association of Xylazine (5mg/kg) and Ketamine (100mg/kg) intraperitoneally, being kept anesthetized throughout the procedure, with maintenance doses of Xylazine (0.4mg/kg) and Ketamine (20mg/kg)^5^.

The surgical procedure consisted of right nephrectomy and subsequential dissection of the vascular pedicle of the left kidney, on which we performed warm ischemia for a period of 20 minutes, with a vascular clamp, followed by renal reperfusion, and then, cystotomy.

The groups were organized as follows: Control Group: with 10 animals, which did not undergo surgical intervention; Sham Group: with eight animals, submitted to the standard surgical procedure and not receiving drugs in the postoperative period; TAC0.1 Group: with 10 animals, undergoing the standard surgical procedure and receiving postoperatively Tacrolimus at a dose of 0.1mg/kg/day; TAC1 Group: with 10 animals, submitted to the standard surgical procedure and receiving postoperative Tacrolimus at a dose of 1mg/kg/day; TAC10 Group: with of 9 animals, undergoing the standard surgical procedure and receiving postoperative Tacrolimus at a dose of 10mg/kg/day; MMF Group: comprising 10 animals, submitted to the standard surgical procedure and receiving postoperative Micophenolate Mofetil at a dose of 20mg/kg/day; MMF/TAC1 Group: with 11 animals, undergoing the standard surgical procedure and receiving postoperative Micophenolate Mofetil at a dose of 20mg/kg/day associated with Tacrolimus at a dose of 1mg/kg/day; and MMF/TAC0.5 Group: comprising 10 animals, submitted to the standard surgical procedure and receiving postoperative Micophenolate Mofetil at a dose of 20mg/kg/day associated with Tacrolimus at a dose of 0.5mg/kg/day.

Three animals died during the experiment, two of them in the immediate postoperative period and one during drug administration (TAC10 Group). All animals received a daily dose of 0.5ml of drug solution and concentrations described with 0.9% saline solution, via gavage with an appropriate catheter and technique, and the animals of the Sham Group received only 0.9% sodium chloride solution.

They were euthanized on the 14^th^ postoperative day, the moment we carried out left nephrectomy and organ preparation for histological analysis.

We sent the blood collected from the animals to the Laboratory of Biochemical Analysis and measured Tacrolimus by the chemiluminescence method, and serum Creatinine, Urea, and Albumin, by the enzymatic colorimetric method of dry chemistry^6^.

We performed the analysis of tissue oxidative stress using kidney samples from each animal that were homogenized and frozen in a tube filled with 2mL of 0.1M phosphate buffer saline (PBS), pH 7.4, with the aid of the BioSpec Tissue-Tearor tissue homogenizer (BIOSPEC PRODUCTS, Bartlesville, USA). We assessed Reduced Glutathione Concentration (GSH)^7^, Lipid Peroxidation (LPO)^8^, and Protein carbonylation (PCO)^9^. All spectrophotometric readings described above were performed on an Infinite^®^ 200 PRO - Tecan^®^ Microplate Reader (Tecan Group Ltd., Seestrasse - Männedorf, Switzerland).

To determine the three-dimensional features of the kidney, we used the stereological method Test System M42. This was superimposed on the histological images to count points and test intersections as described by Mandarim de Lacerda10, at 40x magnification.

We employed a descriptive analysis of the data through tables and graphs. To answer to this work question, we used the ANOVA parametric test with Tukey’s post-test and the non parametric Mann Whitney test. For such analyses, we used the software PRISM 5 for Windows, version 5.0 (GraphPad Software, Inc., 2007). The significance level (α error) adopted was less than 5% (p<0.05).

## RESULTS

After collecting the data, we divided them into two groups for comparison and demonstration of the experiment’s results, as follows:

### 1. Comparative dose-response analysis with the use of tacrolimus

This comparative evaluation aimed at the response of different parameters in relation to logarithmic doses of tacrolimus and its potential renal tissue damage. For this analysis, the groups compared were Control, Sham, TAC0.1, TAC1, and TAC10.

The animals in the Control Group had a mean initial weight of 250g and a mean final weight of 317g (+26.8%). The animals in the Sham Group initially weighed 221g on average, with a mean final weight of 277g (+25.3%). The TAC0.1 Group had an average initial weight of 234g and a final weight of 283g (+21%). The animals in the TAC1 Group had a mean initial weight of 245g and a mean final weight of 295g (+20.4%). The TAC10 Group had an initial weight of 240g and a final weight of 246g (+2.5%).

#### 1.1. Biochemical analysis - using tacrolimus

We observed a difference between the Tacrolimus groups, confirming that it was adequately absorbed during the study period. There was a statistical difference in the serum dosage of the drug in the TAC10 Group, showing a higher concentration of Tacrolimus in the blood of these animals (Table 1).

**Table 1:** Assessment of biochemical parameters - dose/response - using Tacrolimus.

Group	Tacrolimus (ng/ml)	Creatinine (mg/dl)	Urea (mg/dl)	Albumin (g/dl)
Control	AT	0.42 ± 0.14	44.90 ± 2.85*	2.79 ± 0.11
Sham	AT	0.41 ± 0.04	53.75 ± 4.71*#	2.89 ± 0.08*
TAC0 1	0.30 ± 0.19	0.34 ± 0.05	71.00 ± 7.07	2.67 ± 0.22
TAC1	0.49 ± 0.23	0.37 ± 0.05	72.60 ± 6.28	2.90 ± 0.26*
TAC10	2.22 ± 0.74*	0.50 ± 0.05*	94.63 ± 10.81*	2.66 ± 0.22

NOTE: *,# p<0.05 - statistically significant difference; Values presented as mean ± standard deviation.

The serum creatinine values found showed no statistical difference between the Control, Sham, TAC0.1, and TAC1 Groups. The TAC10 Group showed a dosage of 0.50 ± 0.05mg/dl, significantly higher than the other groups (Table 1).

The serum urea dosage, however, was significantly lower in the Control Group than in the other groups, with a value of 44.9 ± 2.85mg/dl. The Sham Group also presented lower values than the others, with 53.75 ± 4.71mg/dl. The TAC1 and TAC0.1 Groups had statistically similar values, while the TAC10 showed a mean dose of 71 ± 7.07mg/dl, significantly higher than the others (Table 1). Serum albumin levels showed statistically similar values between the Control and TAC1 Groups, with values of 2.79 ± 0.11g/dl and 2.90 ± 0.26g/dl, respectively. The Sham Group showed a mean of 2.89 ± 0.08, similar to the TAC1 Group and different from the others. The TAC0.1 and TAC10 groups had similar values, statistically similar to the Control Group and lower than the Sham and TAC1 Groups (Table 1).

#### 1.2. Renal morphometric analysis - using tacrolimus

Regarding renal volumetry, we found that the renal volume was similar between the Control and TAC10 Groups, and between the Sham, TAC0.1, and TAC1 Groups.

There was a statistical difference between the Control and Sham groups, TAC0.1 and TAC1 Groups, and between TAC0.1 and TAC10 Groups. It is worth mentioning that the difference in volume between the TAC1 and TAC10 Groups did not show statistical significance, but p=0.06 showed a trend towards the difference (Table 2). Renal weight was similar between the Control and TAC10 Groups, and between the Sham, TAC0.1, and TAC1 Groups, these being statistically different from the former ones (Table 2).

**Table 2:** Renal morphometry - dose/response - using Tacrolimu.

Group	Renal volume (mm^3^)	Kidney weight (g)
Control	817.5 ± 170.3*	1.2150 ± 0.1446*
Sham	1,240.8 ± 341.1	1.5125 ± 0.1680
TAC0.1	1,211.1 ± 169.4	1.5590 ± 0.1584
TAC1	1,102.2 ± 117.6	1.4510 ± 0.1422
TAC10	931.7 ± 205.9*	1.1575 ± 0.1501*

NOTE: *,# p<0.05 - statistically significant difference; Values presented as mean ± standard deviation.

#### 1.3. Sterological analysis - using tacrolimus

On stereological analysis, we observed no difference in the glomerular volume density (Vvglom) between groups. Likewise, the glomerular number density (Nv) values showed no statistical difference.

#### 1.4. Tissue oxidative stress analysis - using tacrolimus

The biochemical measurements of non-protein thiols (GSH) showed an increased concentration in TAC1 and TAC10 Groups, only the comparison between TAC10 and TAC0.1 being statistically significant.

The concentrations of hydroperoxides (LPO) and protein carbonyls (PCO) showed no significant difference between groups in the assessment of dose response with Tacrolimus.

### 2. Comparative dose-response analysis with association of tacrolimus and mycophenolate mofethyl

This comparative evaluation aimed at the response of different parameters regarding the association of tacrolimus with mycophenolate mofetil and whether the latter can reduce the toxicity of the former at a lower dose. For this analysis, we studied the Groups Control, Sham, TAC1, MMF, MMF/TAC1, and MMF/TAC0.5.

The animals in the Control Group had a mean initial weight of 250g and a mean final weight of 317g (+26.8%). The animals in the Sham Group initially weighed 221g on average, with an average final weight of 277g (+25.3%). The animals in the TAC1 Group had a mean initial weight of 245g and a mean final weight of 295g (+20.4%). The MMF Group showed a mean initial weight of 247g and a final weight of 305g (+23.5%). The MMF/TAC1 Group had a mean initial weight of 254g and a mean final weight of 304g (+19.7%). The MMF/TAC0.5 Group had an initial weight of 248g and a final weight of 303g (+22.2%).

#### 2.1. Biochemical analysis - association of tacrolimus and mycophenolate mofethyl

The samples evaluated for Tacrolimus dosage showed a statistical difference between the TAC1 and MMF/TAC0.5 Groups. The comparison of the MMF/TAC1 and MMF/TAC0.5 Groups had a significance level of p=0.06, demonstrating a trend, despite the lack of statistical difference (Table 3). The serum creatinine values showed no statistical difference between the Control, Sham, and TAC1 Groups, and these had statistically lower values than the MMF, MMF/TAC1, and MMF/TAC0.5 Groups (Table 3). The serum urea dosage showed a statistical difference from the Control Group, with a lower value of 44.90 ± 2.85mg/dl, and TAC1, with a higher value of 72.60 ± 6.28mg/dl. The dosage of the Sham Group was also statistically different from the other groups, except for MMF. The MMF and MMF/TAC1 Groups’ values showed a statistical difference, and there was no difference between them and the MMF/TAC0.5 Group (Table 3). Serum albumin showed no statistical difference between the compared groups, apart from the Sham Group, in which it had significantly higher levels than the Control, MMF, and MMF/TAC1 ones (Table 3).

**Table 3:** evaluation of biochemical parameters - association of tacrolimus and mycophenolate mofethyl.

Group	Tacrolimus (ng/ml)	Creatinine (mg/dl)	Urea (mg/dl)	Albumin (g/dl)
Control	NA	0.42 ± 0.14	44.90 ± 2.85*	2.79 ± 0.11
Sham	NA	0.41 ± 0.04	53.75 ± 4.71*#	2.89 ± 0.08*
TAC 1	0.49 ± 0.23*	0.37 ± 0.05	72.60 ± 6.28*	2.90 ± 0.26
MMF	NA	0.55 ± 0.12*	58.10 ± 7.14*#	2.74 ± 0.21
MMF/TAC1	0.39 ± 0.19	0.57 ± 0.15*	66.64 ± 5.66	2.70 ± 0.24
MMF/TAC0.5	0.24 ± 0.14	0.58 ± 0.13*	61.50 ± 8.96	2.85 ± 0.19

NOTE: *,# p<0.05 - statistically significant difference; NA: Non-aplicable; Values presented as mean ± standard deviation.

#### 2.2. Kidney morphometric analysis - association of tacrolimus and mycophenolate mofethyl

The measured renal volume was statistically lower in the Control Group. The Sham, TAC1, MMF/TAC1, and MMF/TAC0.5 Groups were similar and the MMF Group showed statistically higher values than the TAC1 and MMF/TAC0.5 ones, with no statistical difference from the MMF/TAC1 Group (Table 4).

**Table 4:** renal morphometry - dose/response - association of tacrolimus and mycophenolate mofethyl.

Group	Renal volume (mm^3^)	Kidney weight (g)
Control	817.5 ± 170.3*	1.2150 ± 0.1446*
TAC0.1	1,211.1 ± 169.4	1.5590 ± 0.1584
MMF	1,279.5 ± 107.1*	1.5320 ± 0.2378
MMF/TAC1	1,201.8 ± 247.7	1.5109 ± 0.2823
MMF/TAC0.5	1,139.3 ± 140.9	1.5900 ± 0.1621

NOTE: *, # p<0,05 - statistically significant difference; Values presented as mean ± standard deviation.

### 2.3. Sterological analysis - association of tacrolimus and mycophenolate mofethyl

The quantitative measurement of the glomerular volume density (Vvglom) revealed a statistical difference only between the Control and the MMF/TAC0.5 Groups.

The calculated Vglom of the Sham Group was statistically higher than the others, except when compared with the MMF. The TAC1 and MMF Groups were significantly different, similar to the MMF/TAC1 and MMF/TAC0.5 Groups.

### 2.4. Analysis of tissue oxidative stress - association of tacrolimus and mycophenolate mofethyl

The dosage of non-protein thiols was significantly higher in the MMF Group when compared with the others. Despite not reaching a statistical difference, the comparison between the MMF/TAC1 and MMF/TAC0.5 Groups had a significance level of p=0.06. The concentration of protein carbonyls was smaller in the MMF Group, and this difference was statistically significant, except when compared with TAC1, whose difference had a significance level of p=0.08. The TAC1 and MMF/TAC1 Groups also showed statistical significance in their difference, being similar to MMF/TAC0.5 Group.

## DISCUSSION

FK 506 (Tacrolimus) is a macrolide produced by the fungus Streptomyces tsukubaensis, with potent immunosuppressive activity. It acts on calcium-dependent intracellular biochemical pathways. It then inhibits early T cell activation genes by blocking messenger RNA expression of various cytokines. It belongs to the Group of calcineurin inhibitors (CNI), like cyclosporine, and forms the cornerstone of standard immunosuppressive therapy. Tacrolimus is almost completely metabolized in the liver by the enzyme Cytochrome P450-3A4 (CYP3A4) and eliminated in the feces via the bile^2^.

Mycophenolates are based on Mycophenolic Acid, which inhibits the enzyme inosine monophosphate dehydrogenase (IMPDH). This is a limiting step in the synthesis of guanosine monophosphate (cGMP) in the de novo purine synthesis pathway. As lymphocyte function and proliferation is highly dependent on the de novo purine synthesis, this immunosuppression has a better cytostatic effect on lymphocyte proliferation. The active metabolite of Mycophenolic Acid is metabolized by Glucuronyl Transferase in the liver and most of it is inactively excreted in the urine^1^ ^,^ ^2^ ^,^ ^11^.

Regardless of the therapeutic regimen, immunosuppressive drugs have adverse effects, such as nephrotoxicity, cardiovascular effects, diabetes mellitus, and cosmetic effects such as hirsutism, gingival hyperplasia, and alopecia. In addition, there may also be an influence on the healing process and tissue repair^12^. As for nephrotoxicity, Tacrolimus can exert both a direct cytotoxic effect and ischemic renal cell changes^13^.

Mycophenolate Mofetil does not show a pattern of nephrotoxicity. Thus, recent studies have tried to use it to reduce the dose of other drugs, such as Calcineurin Inhibitors (CNI).

In the case of renal transplantation, there are several immunosuppression regimens. However, in our country the most widely used is an initial association of Tacrolimus and Mycophenolate Mofetil, associated with corticosteroids^1^ ^,^ ^2^.

The study design aimed to evaluate the biological behavior of the kidney in different scenarios of exposure to immunosuppressants commonly used in kidney transplantation. Thus, to quantify renal toxicity against Tacrolimus, a drug known to be nephrotoxic, we used a dose-response evaluation method of drug toxicity, which employs a standardized dose and two other doses, in logarithmic values above and below^14^ ^,^ ^15^. We chose a period of subacute exposure of two weeks, as recommended by Organization for Economic Cooperation and Development (OECD) guideline 407, to prevent unnecessary deaths due to the toxic dose of Tacrolimus and at the same time allow enough time for the end of the inflammatory phase of healing.

Studies have shown that there may be a synergistic effect between immunosuppressants, and thus, there could be a benefit in the combination of drugs to reduce the toxicity of CNI^16^. In this sense, study groups were designed to assess renal toxicity of Tacrolimus and Mycophenolate, and the association of both in the standard and reduced (50%) dose of Tacrolimus.

The period of renal ischemia has been the subject of studies to estimate the ideal time for irreversible lesions not to occur^17^. To this end, there are models of hot^18^ and cold^19^ renal ischemia, and most authors support that hot ischemia of less than 30 minutes is adequate to prevent definitive lesions resulting from hypoperfusion and hypoxia^20^. We used an ischemia time of 20 minutes by clamping the renal vascular pedicle, with the main objective of promoting a perfusion test in the organ and stimulating the organ response to the stress of ischemia and reperfusion, without, however, generating possible permanent injuries that would compromise function.

The evolution of the animals’ weight agrees with the findings of Tomanari, Pine, and Silva^21^, whose animals showed an ascending curve of weight gain until approximately 180 days of life when submitted to an ad libitum diet.

Another nutritional parameter evaluated was the serum albumin level. This is the main serum protein, and is directly related to the bioavailability of Tacrolimus, since the drug’s blood transport occurs mainly linked to albumin and red blood cells ^21^. In the present study, serum albumin values remained stable in all groups, and only the TAC1 Group had slightly higher levels of the protein.

The results found showed different drug concentrations in the TAC0.1, TAC1, and TAC10 Groups, only the dosage in TAC10 being statistically significant, which was significantly higher. When comparing with the drug association groups, the MMF/TAC0.5 Group had lower concentrations than the TAC1 one and tending to be smaller than the MMF/TAC1. The results are consistent with the doses used, as lower doses caused lower serum concentrations.

Regarding Mycophenolate Mofetil, Braun et al.^22^ observed that there may be an increase in its bioavailability when associated with Tacrolimus, though this finding was not confirmed by another study, conducted by Van Gelder et al.^23^.

The last drug administration took place between 24 and 28 hours before blood collection for dosage, justifying the finding that serum concentrations were below the values considered ideal for therapeutic range. Also, the therapeutic window of Tacrolimus is quite narrow and the drug’s toxicity threshold is low, which limits the increase in the administration dose.

When comparing the different doses of Tacrolimus, there was a significant increase in creatinine in the TAC10 Group, which is compatible with the toxicity of the drug at a higher dose. The creatinine values found, however, are within the normal range, between 0.3 and 0.6mg/dl, considered by other researchers and animal facilities^24^ ^,^ ^25^.

There was a progressive increase in urea levels, and for the animals that suffered only renal ischemia (Sham Group) serum urea was higher than the in controls, and from the introduction of the drug the elevation was accentuated, passing to abnormally high levels in the TAC0.1 and TAC1 Groups, and even higher in the TAC10 Group. Castello Branco et al. (2011)^26^ and Spinelli et al. (2012)^27^ consider normal urea values between 40 and 60mg/dl, confirming that in the present study there was an alteration in renal function after the introduction of Tacrolimus, and progressive worsening with higher doses of the drug.

The animals submitted to treatment with Mycophenolate in the different combinations showed higher and borderline levels of serum creatinine. The urea values were also higher for TAC1, MMF, MMF/TAC1, and MMF/TAC0.5 Groups when compared with the Sham Group. This finding demonstrates that the use of Tacrolimus in association with MMF and at a higher dose leads to worsening of renal function.

The renal morphometry measured revealed a compensatory hypertrophy in all groups but TAC10, when compared with the Control one, which remained with both native kidneys. Renal volume showed a gain of 35% to 55% in the Sham, TAC0.1, TAC1, MMF, MMF/TAC1, and MMF/TAC0.5 Groups. Only TAC10 had an average positive variation of 13% in the renal volume. Regarding organ weight, in the same way, only the TAC 10 Group had a 5% reduction. The other groups had a positive variation in organ mass, between 20% and 30%. Santos et al. (2006)^28^ studied the functional and morphological results in rat kidneys undergoing global renal mass reduction, finding an increase of approximately 90% in volume of the remaining kidney eight weeks after contralateral nephrectomy. Seyer-Hansen, Gundersen, and Osterby^29^ studied compensatory renal hypertrophy and found organ weight gain of 31% and 44% after four and 24 days of nephrectomy, respectively.

Therefore, the present study demonstrated that compensatory renal hypertrophy was not affected by immunosuppressive medications in the short term, except in the case of high doses of Tacrolimus, which demonstrates a direct nephrotoxic effect in this scenario. Such findings are comparable to those found by the aforementioned authors.

With the glomerular analysis by stereology, there was no glomerular sclerosis or fibrosis/sclerosis of the Bowman’s capsule, which represent classic alterations of chronic renal toxicity related to CNI^13^.

The measurement of glomerular volume density and glomerular numerical density found was similar between the response assessment groups, and the calculation of mean glomerular volume showed a difference between the Sham Group compared with Control, TAC0.1, and TAC1 Groups. This finding, together with the greater compensatory renal hypertrophy in the Sham Group, suggests that the use of Tacrolimus, even at a low dose, affects glomerular hypertrophy after contralateral nephrectomy. Likewise, when the drug association groups were compared, there was a statistical difference in the glomerular volume density between the Control and MMF/TAC0.5 Groups, and there was no difference between the groups when the glomerular number density was compared. The calculated mean glomerular volume showed a greater compensatory hypertrophy in the Sham and MMF Groups, suggesting, in addition to a deleterious effect of Tacrolimus, a possible protective effect of Mycophenolate Mofetil regarding the increase in volume of the glomeruli in compensatory renal hypertrophy, since the animals in the MMF Group had the largest renal volume.

Although the data found in this study did not clearly demonstrate a great difference between the groups, they agree with the findings in the literature. Another point of discussion is that perhaps stereology or even pure histomorphological assessment are not sufficient to accurately assess changes related to nephrotoxicity in a model of subacute drug exposure, as in this experiment.

The results found showed a progressive increase in the concentration of GSH in the dose-response curve when the dose of Tacrolimus was increased, although we found a statistical difference only in the comparison of 10mg/kg/day with 0.1mg/kg/day. This must have occurred because the time of exposure to the drug was not long enough. When comparing the drug association groups, there was a significant increase in GSH levels in the MMF Group. Sarangi et al.30 reported an increase in GSH in rats treated with Mycophenolate after mercury intoxication, suggesting that such an increase is a protective factor for the cell. Likewise, Saad, Arafah, and Najjar^31^ point out that increased GSH is one of the cellular mechanisms to combat oxidative stress at the cellular level.

The concentration of hydroperoxides and protein carbonyls showed no statistical difference when comparing the dose-response groups to Tacrolimus, inferring that there was no significant lipid peroxidation or protein carbonylation in these groups. Regarding the comparison of drug association, however, MMF and MMF/TAC0.5 Groups showed a significant reduction in the concentration of hydroperoxides when compared with the others, thus implying lower lipid peroxidation. Similarly, the concentration of protein carbonyls was statistically lower in the MMF Group, and there was also a difference between the TAC1 and MMF/TAC1 Groups, suggesting less oxidative damage to proteins in the pure Mycophenolate Group and a synergistic effect of Tacrolimus and Mycophenolate increasing the protein carbonylation when compared with drugs alone.

This study allows us to conclude that: a) The evaluation of renal function by measuring creatinine and urea showed nephrotoxicity related to the use of Tacrolimus, especially at high doses, resulting in greater changes in urea levels than in creatinine ones; similarly, there is an alteration in renal function with the use of Mycophenolate Mofetil, and there is a worsening of renal function in the combination of drugs; b) High dose Tacrolimus inhibits compensatory renal hypertrophy; there is no glomerular histological change in this time period of drug exposure; Glomerular stereology showed inhibition of compensatory glomerular hypertrophy against drugs, when the mean glomerular volume was evaluated; c) The drugs induced a protective cellular reaction against oxidative stress through the increase of non-protein thiols (GSH), with no alteration in lipid peroxidation or significant protein carbonylation detected with the use of Tacrolimus, even at high doses; Mycophenolate Mofetil may be a protective factor against lipid peroxidation and protein carbonylation.

**LIST OF ACRONYMS**
ABTO : - Brazilian Association of Organ Transplantation
PUFA : - Polyunsaturated fatty acids
BSA : - bovine serum albumin
CEUA : - Ethics Committee in the Use of Animals
CNI : - calcineurin inhibitor
COBEA : - Brazilian College of Animal Experimentation
DTNB : - 5,5’-dithio-bis-2-nitrobenzoic acid
ROS : - Reactive Oxygen Species
FeSO4NH 4 : - ferrous ammonium sulfate
FK 506 : - Tacrolimus
GSH : - Reduced Glutathione
HCl : - hydrochloric acid
HLA : - Human Leukocyte Antigen
LPO : - Lipid Peroxidation
MTOR : - Mammalian Target of Rapamycin
PBS : - Buffer phosphate saline
PCO : - Protein carbonylation
PTK : - Protein Tyrosine kinase
TCA : - Trichloroacetic acid
TCR : - T cell receptor
